# Outcomes of concomitant midurethral sling and anterior colporrhaphy in managing stress urinary incontinence associated with cystocele: a systematic review and single-arm analysis

**DOI:** 10.1007/s00345-025-05665-7

**Published:** 2025-07-30

**Authors:** Mohamed Tharwat, Reham Ramadan, Mohamed Abd-ElGawad, Abdelwahab Hashem, Diaa-Eldin Taha

**Affiliations:** 1 Urology department, Almoqataah Central hospital, Dakahlia, Egypt., Urology department, King Salman Armed Forces Hospital, Tabuk city, KSA., Dakahlia, Tabuk, Egypt; 2 Egyptian Ministry of Health, Dakahlia, Egypt; 3 Faculty of Medicine, Fayoum University, Fayoum, Egypt; 4 Urology department, Faculty of medicine, Delta University for Science and Technology, Dakahlia, Egypt; 5 Urology department, Faculty of Medicine, Kafrelsheikh University, Kafr El Sheikh, Egypt

**Keywords:** Stress urinary incontinence, Cystocele, Midurethral sling, Anterior colporrhaphy, Anterior vaginal wall prolapse, Systematic review

## Abstract

**Background:**

Multiple surgical approaches have been provided for treating Stress urinary incontinence (SUI) combined with anterior vaginal wall prolapse (cystocele). However the optimal treatment remains a topic of ongoing debate. This systematic review and single-arm analysis aimed to evaluate the efficacy and safety of midurethral sling (MUS) with concomitant anterior colporrhaphy (AC) for surgical treatment of stress urinary incontinence associated with grade 1–3 anterior vaginal wall prolapse.

**Methods:**

We searched through multiple databases including, PubMed, Cochrane, Scopus, and Web of Science using a well-established search strategy to identify studies assessing the outcomes of MUS with concomitant AC in women with SUI and grade 1–3 cystocele. Data was extracted from the relevant studies in a predefined excel sheet. Data analysis was accomplished by using OpenMeta [analyst] software.

**Results:**

We found 744 articles and included eight studies in the analysis. The pooled objective cure rate of SUI was 0.907 (95% CI 0.871 to 0.943), objective failure of SUI was 0.036 (95% CI 0.002 to 0.071), the subjective cure rate was 0.825 (95% CI 0.717 to 0.932), and the anatomical success rate for cystocele was 0.919 (95% CI 0.851 to 0.987). De novo urge urinary incontinence was reported in 12 of 81 patients, and four of 46 patients developed de novo OAB symptoms. Postoperative retention was noted in 5% of 405 patients. Long-term follow-up data from two studies indicated a durable effect.

**Conclusion:**

MUS combined with AC is an effective option for treating SUI associated with grade 1–3 cystocele, with high rates of both objective and subjective cure.

## Introduction

Stress urinary incontinence (SUI) is a serious condition defined as uncontrollable urine leakage that occurs when bladder pressure is higher than urethral closure pressure related to coughing, sneezing, physical activity, or exercise. It is considered the most prevalent type of urinary incontinence [1]. Intrinsic sphincter deficiency (ISD) or urethral hypermobility may cause stress urine incontinence; however, both disorders may occur simultaneously [2]. SUI is not a condition that threatens lives; however, this problem has serious physical, psychological, and social effects for individuals. It may lead to social isolation and a reduced quality of life in affected patients [3].

It is frequent for SUI and pelvic organ prolapse (POP) to exist simultaneously. It is reported that nearly 40–63% of individuals with SUI have POP, whereas 55% of patients with POP have SUI [4]. Approximately 11% of women may need surgery for both SUI and POP [4]. Anterior vaginal wall prolapse is considered the most prevalent type of POP. It is also known as cystocele. Repairing multiple defects at the same operation is desirable to decrease anesthesia risk and to provide the patient with one recovery period [5, 6]. It is challenging to provide proper surgical management for both SUI and massive prolapse [7].

Although multiple surgical approaches have been introduced for treating SUI and/or POP, we lack convenient procedures that are capable of repairing both dysfunctions with a high success rate and a low rate of recurrence [8]. The polypropylene mesh mid-urethral sling is widely regarded as the standard treatment for SUI by authoritative bodies in the fields of urodynamics and urogynecology [9]. Although the mid-urethral sling (MUS) is a beneficial choice for individuals with SUI, it involves risks, even at experienced facilities participating in the Mid-Urethral Sling Trial (TOMUS) [10].

Many centers consider the anterior colporrhaphy (AC) as the standard approach for cystocele repair, with or without sling placement [11]. Each year, over 300,000 pelvic organ repair surgeries are carried out in the United States [12], with AC occupying 81% of them [4]. Forty percent or more of patients may experience a recurrence of anterior vaginal prolapse following conventional anterior colporrhaphy [13–15]. According to the theories of Petros and Delancy, it has been noted that the pelvic floor contains fixing points that the operator should look for to stop the anterior vaginal wall from descending. This concept leads to the use of polypropylene tapes to support the urethra and suggests that they may provide enough support for both the anterior vaginal wall and the base of the bladder above it [16–18].

As we stated before, it is crucial to realize that SUI is not only a urethral condition caused by intrinsic sphincter deficiency; urethral hypermobility can be an extra cause, which results from the laxity of the anterior vaginal wall. Therefore, to treat stress urinary incontinence efficiently, anterior vaginal wall support is essential. Consequently, a midurethral sling (MUS) combined with anterior vaginal wall repair may improve the correction of SUI [19, 20].

Despite the widespread use of midurethral sling (MUS) and anterior colporrhaphy (AC), most of published studies discuss either MUS or AC, with minimal research examining the combination of the two interventions. These studies discussed the efficacy of both interventions together, but there is a huge variation in the reported cure rate. Some studies reported a cure rate of 80.5% [21], while other studies reported high cure rate of 97% [22]. Therefore a thorough evaluation of this combined strategy is required to better understand its efficacy in the treatment of SUI and cystocele repair.

This systematic review and single arm met-analysis aims to assess the effectiveness of concomitant midurethral sling (MUS) and anterior colporrhaphy (AC) for treating SUI associated with cystocele. By analyzing the mean cure rate from all studies combining both interventions, we seek to determine a pooled global cure rate, which may provide more specific clinical practice guidance.

## Materials and methods

### Study design

This systematic review and single-arm meta-analysis was carried out in accordance with the guidelines outlined in the updated Preferred Reporting Items for Systematic Reviews and Meta-Analyses (PRISMA) statement and the Cochrane Handbook for Systematic Reviews of Interventions [23, 24].

The systematic review registration number CRD42024584771 was secured from PROSPERO, the international prospective register of systematic reviews managed by the National Institute for Health Research.

We conducted a methodical search across major online databases, including Scopus, Cochrane Library, PubMed, and Web of Science. We searched from 2001 to June 2024 using relevant keywords, with no restrictions applied. We provided the search strategy that we used in supplementary file 1.

### Inclusion criteria

All studies fitting each of the following requirements were included: Female patients with SUI and grades 1–3 cystocele represented the study population.

The intervention was MUS procedures as the therapeutic approach for treating SUI, in conjunction with AC for the correction of cystocele. Studies that report sufficient and reliable data about the cure rate of SUI (e.g., urodynamic studies, pad test, stress test) as our primary outcome. It involved data about the success rate of cystocele, and postoperative complications as our secondary objective. We included the original articles such as, randomized controlled trials (RCTs), cohort studies, as well as non-randomized controlled trials with no restriction on date of publication to include all relevant studies.

### Exclusion criteria

1. Studies which does not provide prober data for extraction and analysis. 2. Review articles, books, editorials, thesis, commentaries, and conference abstracts. 3. Non English articles. 4. Female patients’ under18 years. 5. Patients with grade 4 cystocele, as they typically require more complex treatments, which differ from those for grade 1–3 cystoceles. Including these cases could introduce clinical variability and affect the results of the meta-analysis. 6. Any intervention for treating SUI and cystocele other than MUS with concomitant AC.

An initial screening of the title and abstract of the retrieved studies was done by two authors. Two authors initially screened the titles and abstracts of the retrieved studies, and then evaluated the full texts of the relevant studies on an excel sheet to determine their eligibility. The third author was consulted to settle any disputes. Data extraction was not feasible as the studies included other interventions for prolapse repair (posterior colporrhaphy, with or without anterior colporrhaphy or hysterectomy) without differentiating the outcomes between the various procedures.

### Date extraction and study outcomes

Two independent authors extracted data from the relevant studies in a predefined excel sheet including, summary, baseline, and outcomes sheets. The summary sheet involved the following parameters, study ID, country, site involved, and time of realization, study design, sample size, inclusion criteria, exclusion criteria, the type of mid-urethral sling used in treatment, (MUS and Anterior colporrhaphy) group, preoperative parameters, outcomes, result, follow up, and additional notes if present.

The parameters included in baseline table were study ID, number of the patients, age, BMI, parity, vaginal deliveries, postmenopausal, preoperative urodynamic parameters Maximum flow rate (Q max), post voiding residual urine (PVR), and anterior vaginal wall prolapse stage.

"The outcome sheet included the sample size. It also recorded the objective cure rate of SUI, categorized as cure, improvement, failure, and scale. The subjective cure rate of SUI was documented as cure, improvement, and failure.

Postoperative urodynamic parameters were included, such as Q max, change in Q max, post-void residual volume (PVR), change in PVR, maximum cystometric capacity (MCC), detrusor pressure at maximum flow (DPMF), first desire to void (ml), maximum urethral closure pressure (MUCP), and urethral functional length (UFL).

Postoperative complications were noted, including frequency, urgency, bladder injury, urgency incontinence, difficulty emptying the bladder, de novo urge urinary incontinence, de novo overactive bladder (OAB) symptoms, urinary tract infection, hematoma, postoperative retention, residual urine volume (> 50 ml), dyspareunia, tape complications, and de novo urgency. It also reported cystocele repair, categorized as anatomical success or failure. Additional notes were included if present. Discrepancies identified during the data extraction process were addressed and resolved through consultations among the authors.

### Outcomes definition

The objective cure rate of SUI was the main outcome of our systematic review and single-arm analysis. A patient was considered cured if there was no urinary leakage during the pad test or the cough stress test, if the pad weight was less than 1 g, or if there was no need for pad use. This outcome was evaluated using various measures, including urodynamic studies, pad tests, and stress tests.

Our secondary outcomes included objective failure of SUI, defined as no change between preoperative and postoperative findings on the cough stress test or the pad test, Subjective cure of SUI, success cure of cystocele, and postoperative complications, including postoperative urinary retention, urinary tract infection, tape complications, and de novo urge incontinence.

The subjective cure of SUI was determined as the absence of urine loss during daily activities, based on patients'self-reported outcomes.

The Success cure of cystocele was referred as anterior vaginal wall prolapse stage ≤ 1 according to the pelvic organ prolapse quantification (POP-Q) system, absence of the vaginal wall descent beyond the hymen, or prolapse retreatment during the study.

### Risk of bias and quality of included studies assessment

The risk of bias for the retrieved studies was evaluated by two independent reviewer utilizing the Cochrane Risk of Bias Tool for Randomized Trials (ROB 2) [23]. The ROB 2 tool evaluates risk of bias across six aspects: the process of randomization, deviations from planned interventions, handling of missing outcome data, measurement of outcomes, selection of reported results, and other potential biases. The reviewers'judgments are classified as yes, probably yes, probably no, or no information [23].

The Newcastle–Ottawa scale (NOS) was employed to evaluate the quality of observational studies [25]. NOS tool consists of the following domains, Selection (representativeness, selection, exposure ascertainment, outcome absence at start), Comparability (cohort comparability by design/analysis), as well as Outcome (outcome assessment, follow-up duration, follow-up adequacy). Reviewers answered NOS by awarding stars for each criterion within the three domains: Selection (with a maximum of 4 stars), Comparability (with a maximum of 2 stars), and Outcome (with a maximum of 3 stars). We assessed the quality of evidence using the GRADE approach, considering factors such as risk of bias, consistency, indirectness, publication bias, and imprecision [26]. We resolved any disputes through discussion [25].

### Data analysis

Statistical analysis was conducted utilizing Open Meta[analyst]. The present meta-analysis estimated proportions or rates for dichotomous data, and the pooled effect size for continuous data using mean. Both were with 95% confidence intervals (CIs). We assessed the heterogeneity by using p- value and I-square test. Studies with a p-value below 0.1 are regarded as exhibiting significant heterogeneity.

We applied a random—effect model due to the heterogeneous nature of the studies included. Additionally, we conduct a Leave-One-Out sensitivity analysis, where each study is sequentially excluded to assess its influence on heterogeneity and the total effect estimate.

## Results

### Data collection and study selection

**Fig. 1:**
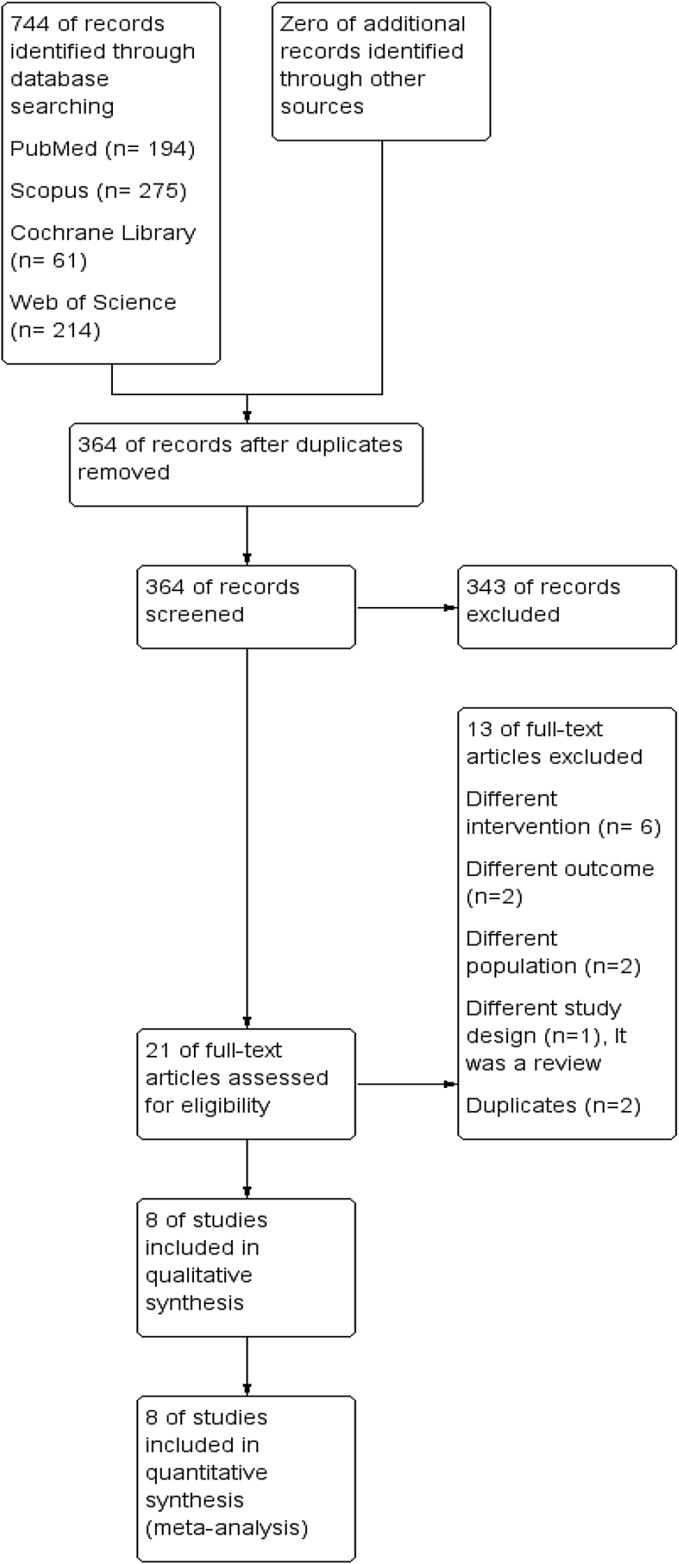
PRISMA flow diagram illustrating the study selection process: identification, screening, eligibility, and inclusion. It shows the number of records identified, screened, excluded, and the final studies included in the systematic review. Each stage represents the flow of studies through the review process

A total of 744 articles were obtained from the electronic database search. After removing the duplicates, title and abstract review was performed in the remaining 364 articles, after which 21 underwent full text screening. We excluded six studies because they involved different interventions or it was not possible to extract the data as the studies included other interventions for prolapse repair without differentiating the outcomes between the various procedures. Two studies did not report any outcomes of interest, two studies had a different population, two studies were duplicates, and one study was a review. Finally, Only 8 studies were included in the synthesis. Figure 1 is used to illustrate the entire study selection process.

In this systematic review, 499 patients diagnosed with SUI combined by grade 1–3 cystocele underwent midurethral sling (MUS) surgery with concomitant anterior colporrhaphy across the included studies. We provided a summary of the included studies Table 1. The lowest mean age of the patients in our systematic review was 43.19, and the highest was 66. The preoperative mean value of Qmax was in the range of 17.23 up to 28.4. Baseline characteristics and preoperative urodynamic parameters are provided in Table 2.

**Table 1:** Summary of the included studies

Study ID	Country, Site involved, and time of realization	Study design	Sample size (00 patients)	Sample size of the (MUS and Anterior colporrhaphy) group (00 patients)	Result
Abdel Aziz et al. 2020	Egypt, Urology Department, Faculty of Medicine, Cairo University, between June 2014 and April 2016	Randomized control trail	72	36	• The cure rate of group 1 was 66.7%, while for group 2 it was 90% after 12 months (p < 0.05) • Six patients (17%) with asymptomatic stage II cystocele in group 1 became symptomatic or developed higher stage after 12 months that required surgical repair
Liapis et al. 2010	Greece, the 2nd Department of Obstetrics and Gynecology of the University of Athens, At the beginning of 2008	Prospective follow-up observational study	121	41	• The objective cure rate for the patients who had undergone TVTO only was 81% (57/70); when considering patients lost to follow-up as clinical failure results the objective cure rate was 77% (57/74) • The objective cure rate of USUI for patients who underwent TVTO and cystocele repair was 84% (32/38) • Regarding cystocele management, the success rate was 92% (35/38)
Reich-2007	Germany, Department of Gynecology and Obstetrics, University of Ulm, between January 2001 and December 2003	Prospective observational study	284	40	• The objective cure rate was 90% in women after isolated TVT procedures, 97% after TVT procedures and anterior colporrhaphy, and 85% in patients after TVT and posterior colporrhaphy. Improvement of stress incontinence was found in 9%, in 3%, and in 12% respectively
Liapis-2001	Greece, the 2nd Department of Obstetrics and Gynecology of the University of Athens	Prospective study	68	18	• 90% success rate was achieved at 24 months’ follow-up for patients with stage I cystocele, while the success rate of the TVT procedure in patients with anterior coloporrhaphy and TVT was 88.8% at 24 months’ follow-up
Zullo et al. 2008	Italy, Department of Obstetrics and Gynecology, University of Rome ‘‘Campus Bio-Medico,’’ From June 2004 through May 2006	Pilot study	50	50	• In all, 43 (91%) and 46 (92%) patients were objectively cured for cystocele and SUI, respectively. Overall early postoperative complication rate was 16%, although all were minor • Only 1 patient, at 12-month follow-up, developed tape erosion that required surgical removal
Ahmed et al. 2020	Egypt, Department of Urology, Faculty of Medicine, Al-Azhar University, from June 2015 to September 2018	Prospective randomized trial	84	41	• At the end of study, the SUI cure rate was 85.7% in the total study cohort, 82.9% • POP was successfully repaired in 91.7% of the total study cohort, 85.4%
yonguc et al. 2014	"Turkey, Department of Urology, Izmir Bozyaka Training and Research Hospital, from March 2005 to March 2010."	Retrospective cohort study	226	226	• Our overall complication rates were 24 and 6.1% in groups 1 and 2, respectively. Postoperative urinary retention (UR) occurred in nine women (11.3%) in group 1, which required an indwelling catheterization for 7–14 days • There was no UR in group 2
Lau et al. 2011	Taiwan, Department of Gynecology and Obstetrics, Taipei Veterans General Hospital, From April 2007 to January 2008	Retrospective study	115	47	• The short-term (1 year) objective cure rates and failure rates for cystocele were 98.5% vs. 86.9% (P = 0.018) and 1.5% vs. 13.0% (P = 0.018), respectively, in Group I and Group II

**Table 2:** Baseline characteristics and preoperative urodynamic findings of the patients

Study ID		Number of the patients	Age (years) mean &SD	BMI (Body mass index) mean &SD	Parity mean &SD	Q max (Maximum flow rate, ml/s) mean &SD	PVR (postvoiding residual urine, ml) mean &SD	Anterior vaginal wall prolapse stage
Abdel Aziz et al. 2020	MUS and Anterior colporrhaphy	36	52 ± 9.4	30 ± 3.1	*	23.5 ± 10.2	40.1 ± 24.4	Asymptomatic stage II anterior compartment vaginal wall prolapse (cystocele)
Liapis et al. 2010	MUS and Anterior colporrhaphy	41	57.1 ± 10.6	27.1 ± 1.9	2 ± 0.96	20.54 ± 3.94	18.46 ± 20.45	Stage II prolapse of the anterior compartment
Reich et al. 2007	MUS and Anterior colporrhaphy	40	66	29.3	*	*	*	Diagnosed with cystocele without specifying the grade of the cystocele
Liapis et al. 2001	MUS and Anterior colporrhaphy	18	54.2 ± 8.1	27.2 ± 3.3	2.1 ± 1.3	*	*	Stage II prolapse of the anterior compartment
Zullo et al. 2008	MUS and Anterior colporrhaphy	50	63 ± 7.5	23.25 ± 2.6	2.25 ± 1.46	28.4 ± 6.8	28 ± 10	Stage 1 3 patients (6%) Stage 2 22 patients (44%) Stage 3 25 patients (50%)
Ahmed et al. 2020	MUS and Anterior colporrhaphy	41	43.19 ± 7.91	28.39 ± 3.77	3.24 ± 1.32	24.56 ± 7.67	8.78 ± 12.83	Stage 2 and 3 cystocele
yonguc et al. 2014	MUS and Anterior colporrhaphy	79	52.7 ± 9.1	29 ± 2.6	*	*	*	Stage 2 or stage 3 anterior wall prolapse [according to the Pelvic Organ Prolapse Quantification (POP-Q) system]
	MUS and Anterior colporrhaphy	147	51.8 ± 9.3	28.7 ± 2.6	*	*	*	
Lau et al. 2011	MUS and Anterior colporrhaphy	47	57 ± 12.68	*	3.1 ± 1.76	17.23 ± 9.4	12.68 ± 41.18	37 patients with stage 2, 9 patients with stage 3, and 1 patient with stage 4 according to POP-Q stage

* *p*=0.05

### Quality assessment results

**Fig. 2:**
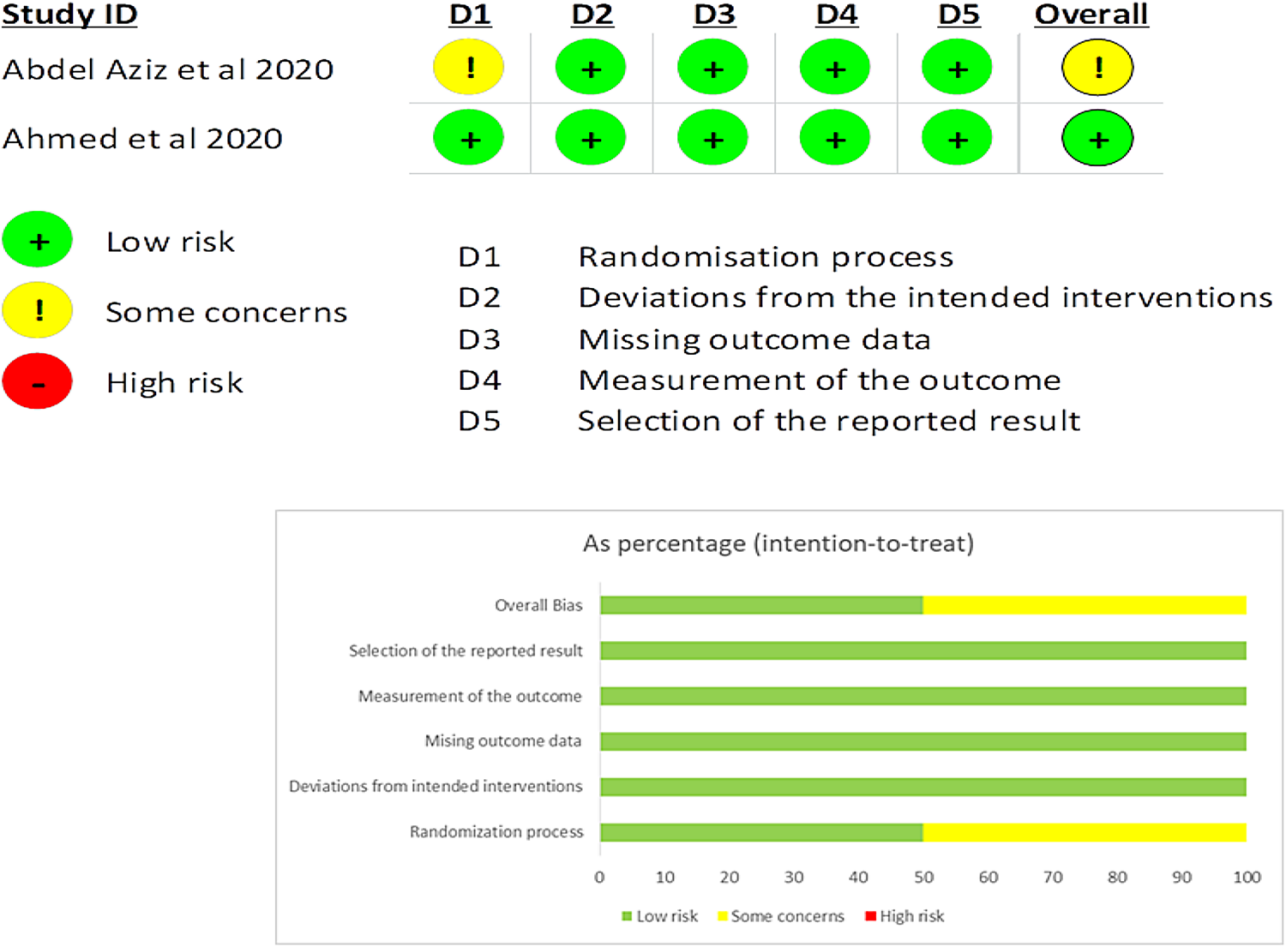
Quality Assessment Using Cochrane Risk of Bias 2 (ROB 2) Tool

The Cochrane Risk of Bias 2 (ROB 2) tool was utilized to evaluate the randomized clinical trials in our review. A single study was evaluated, revealing a low risk of bias [27]. However, Abdel Aziz et al. 2020 was assessed as having some concerns because of insufficient data on allocation concealment and blinding of participants [28]. The quality assessment summary is provided in (Fig. 2). With the exception of a single study, Liapis et al. 2001 [29], which was rated as poor quality due to its failure to provide detailed baseline characteristics for each group, which is essential for ensuring comparability, the general quality of the cohort studies was determined to be good using NOS Table 3.

**Table 3:** Quality assessment, Newcastle–Ottawa scale (NOS) for observational studies

	Selection				Comparability	Outcome			Quality score
Study ID	Representativeness of Exposed Cohort	Selection of Non-Exposed Cohort	Ascertainment of Exposure	Demonstration that outcome of interest was not present at start of study	Comparability of cohort on the basis of the design or analysis	Assessment of Outcome	Was follow-up long enough for outcomes to occur	Adequacy of follow-up of cohorts	
Liapis et al. 2010	*	*	*	*	**	*	*	*	Good
Reich et al. 2007	*	*	*	*	**	*	*	*	Good
Liapis et al. 2001	*	*	*	*		*	*	*	Poor
yonguc et al. 2014	*	*	*	*	**	*	*	*	Good
Lau et al. 2011	*	*	*	*	**	*	*	*	Good

A maximum of **one star** can be awarded for each item within the **Selection** and **Outcome** categories. A maximum of **two stars** can be awarded for **Comparability. Selection**: Assesses representativeness of the exposed cohort, selection of the non-exposed cohort, ascertainment of exposure, and whether the outcome was present at the start of the study. **Comparability**: Assesses the control for important factors in the study design or analysis, with a maximum of **two stars** for this category. Outcome: Assesses the assessment of outcomes, follow-up duration, and adequacy of follow-up.

The GRADE ratings showed moderate quality for objective cure rate, postoperative retention, and anatomical success of cystocele, with concerns about indirectness and publication bias. Evidence for objective failure of SUI and tape complications was low due to imprecision and publication bias [26]. Table 4.

**Table 4:** The quality of evidence was assessed using the GRADE approach

Outcome	Study design	No of studies	No of participants	Risk of Bias	Inconsistency	Indirectness	Imprecision	Publication bias	Quality of evidence	Effect estimate	Importance
Objective cure rate of SUI	RCTs and Observational studies	8	498	Not Serious	Not Serious	Serious (−1)	Not Serious	Serious (−1)	● ● ● ○ (Moderate)	0.907 (0.871 to 0.943)	Critical
Objective failure of SUI	RCT and Observational studies	5	186	Not Serious	Not Serious	Serious (−1)	Serious (−1)	Serious (−1)	● ● ○ ○ (Low)	0.036 (0.002 to 0.071)	important
Subjective cure rate of SUI	Observational studies	4	325	Not Serious	Not Serious	Serious (−1)	Not Serious	Serious (−1)	● ● ○ ○ (Low)	0.825 (0.717 to 0.932)	important
Postoperative retention	RCT and Observational studies	5	405	Not Serious	Not Serious	Serious (−1)	Not Serious	Not Serious	● ● ● ○ (Moderate)	0.050 (0.025 to 0.076)	Critical
Postoperative urinary tract infection	RCT and Observational studies	4	179	Not Serious	Not Serious	Serious (−1)	Serious (−1)	Serious (−1)	● ● ○ ○ (Low)	0.048 (0.017 to 0.079)	important
Anatomical success of cystocele	RCTs and Observational studies	4	173	Not Serious	Not Serious	Serious (−1)	Not Serious	Serious (−1)	● ● ● ○ (Moderate)	0.919 (0.851 to 0.987)	Critical
Tape Complications	RCT and Observational studies	7	458	Not Serious	Not Serious	Serious (−1)	Serious (−1)	Serious (−1)	● ● ○ ○ (Low)	0.007 (−0.000 to 0.015)	important

### Primary outcome

**Fig. 3:**
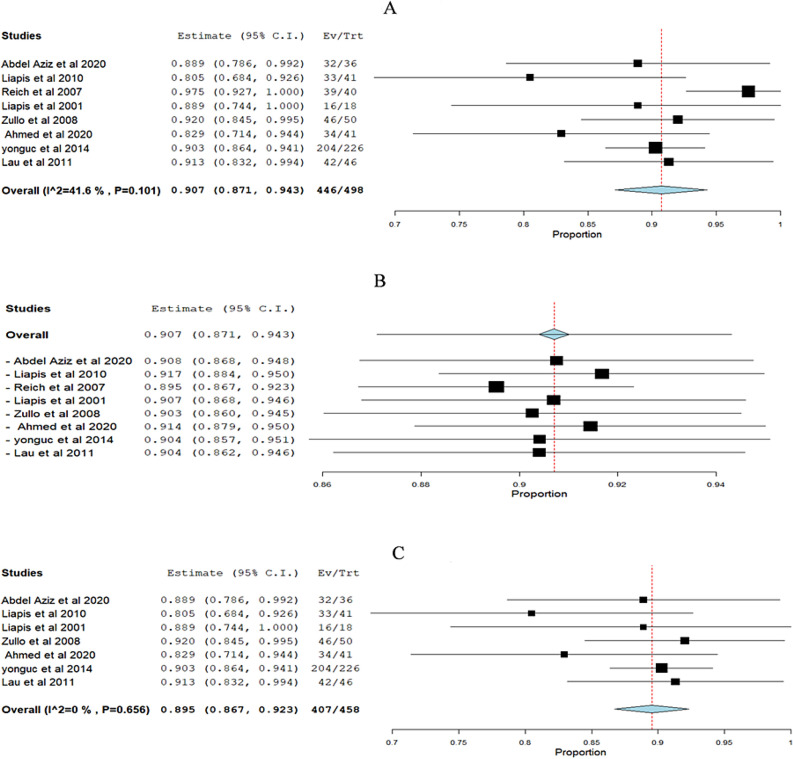
Meta-analysis forest plots for the objective cure rate of stress urinary incontinence (SUI): **A** Pooled cure rate with moderate heterogeneity (I^**2**^ = 41.6%, p = 0.101). **B** Leave-one-out sensitivity analysis. **C** Pooled cure rate with no heterogeneity (I^**2**^ = 0%, p = 0.656). Squares represent individual study estimates; diamonds show overall effect sizes

**Objective cure rate of (SUI):** The overall pooled effect estimate from eight studies was 0.907 (95% CI 0.871 to 0.943). The studies showed a heterogeneity, with an I^2^ value of 41.6% and a corresponding p-value of 0.101 Fig. 3A. We performed a leave-one-out analysis by excluding Reich et al. 2007 [22], which resulted in the p-value for heterogeneity increasing to 0.656 and the I^2^ reducing to 0% Fig. 3B, C.

### Secondary outcomes

**Fig. 4:**
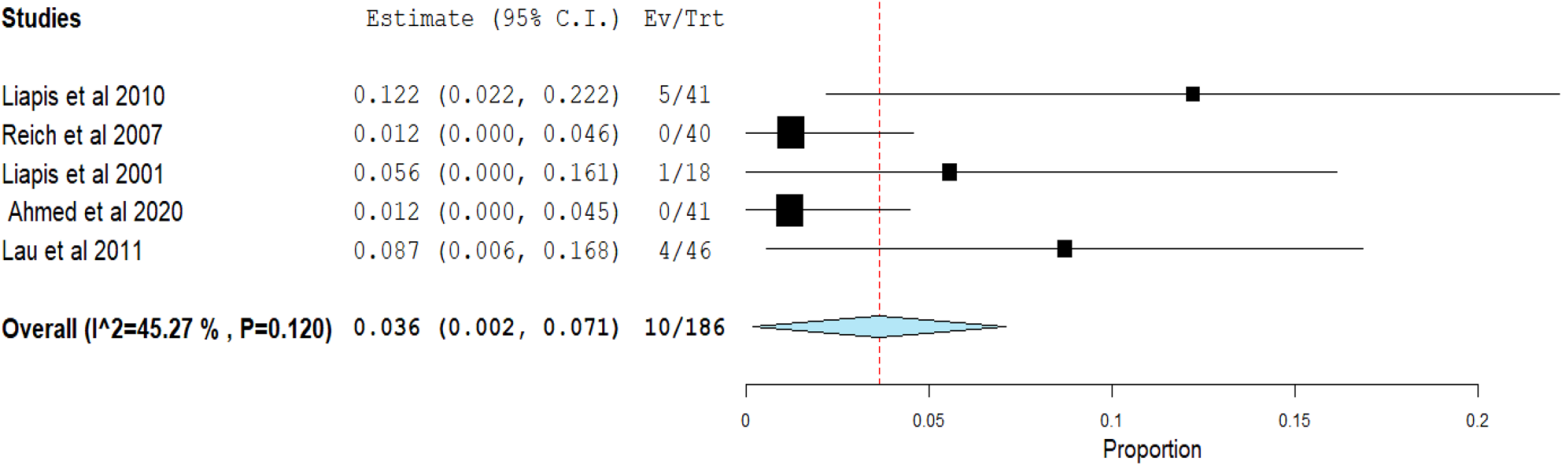
Meta-analysis forest plots for the Objective failure of stress urinary incontinence (SUI): Forest plot showing the pooled estimate (0.036; 95% CI 0.002–0.071) for objective failure of SUI with moderate heterogeneity (I^**2**^ = 45.27%, p = 0.120). Squares represent individual studies; the diamond shows the overall effect size

**Objective failure of SUI:** The overall pooled effect estimate from five studies was 0.036 (95% CI 0.002 to 0.071). With a p-value of 0.120 and an I^2^ of 45.27%, the studies did not exhibit statistically significant heterogeneity (Fig. 4).

**Fig. 5:**
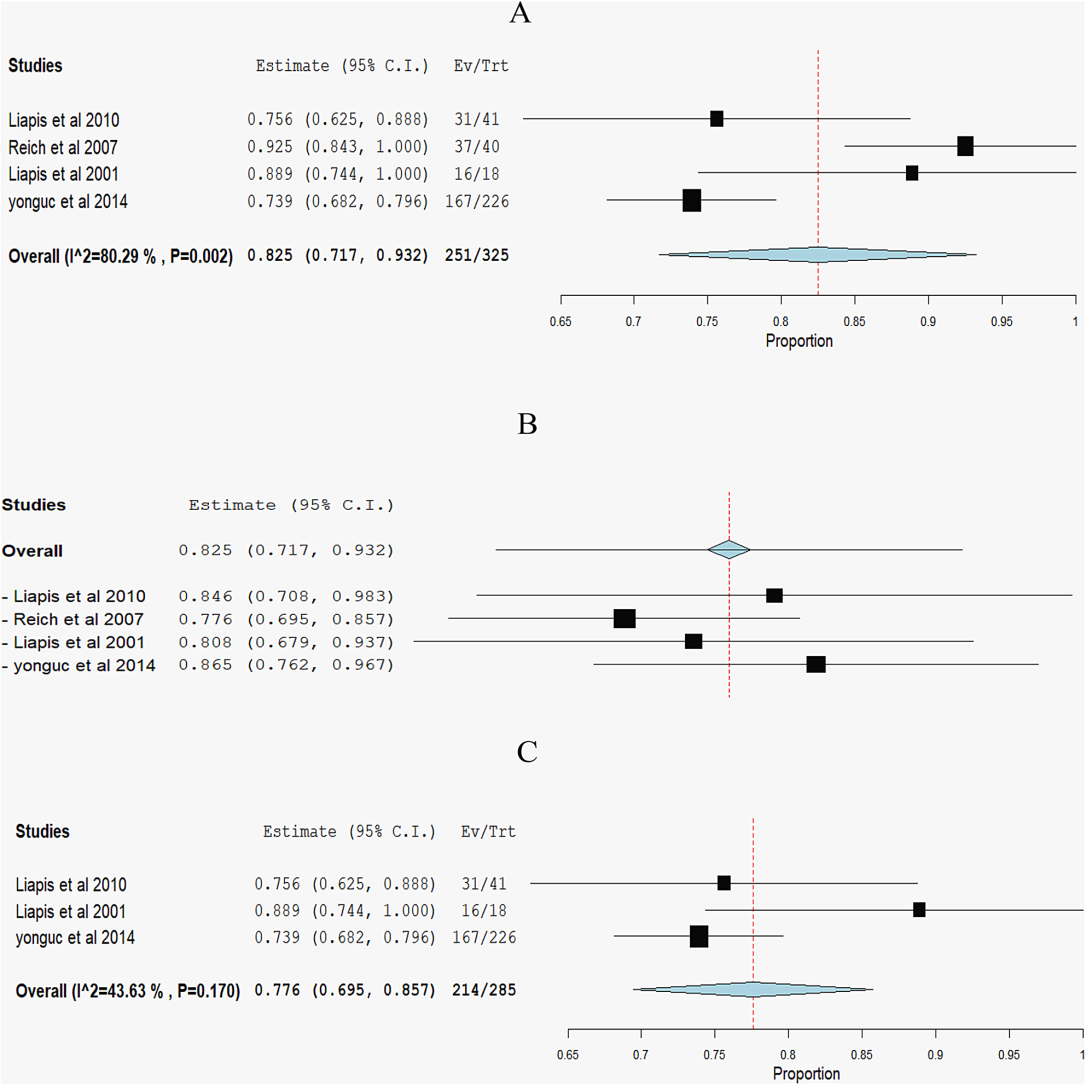
Forest plots for the subjective cure rate of stress urinary incontinence (SUI): **A** Pooled estimate with high heterogeneity (I^**2**^ = 80.29%, p = 0.002). **B** Leave-one-out sensitivity analysis. **C** Pooled estimate with moderate heterogeneity (I^**2**^ = 43.63%, p = 0.170). Squares represent individual studies; diamonds indicate overall effect sizes

**Subjective cure rate of SUI:** The overall pooled effect estimate from four studies was 0. 825 (95% CI 0.717 to 0.932). The analysis revealed substantial heterogeneity, indicated by an I^2^ of 80.29% and a corresponding p-value of 0.002 Fig. 5A. In order to tackle the heterogeneity issue, we performed a leave-one-out sensitivity analysis Fig. 5B. Excluding Reich et al. 2007 [22] effectively solved the heterogeneity, resulting in the p-value for heterogeneity rising to 0.17 and I^2^ reducing to 43.63% Fig. 5C

**Fig. 6:**
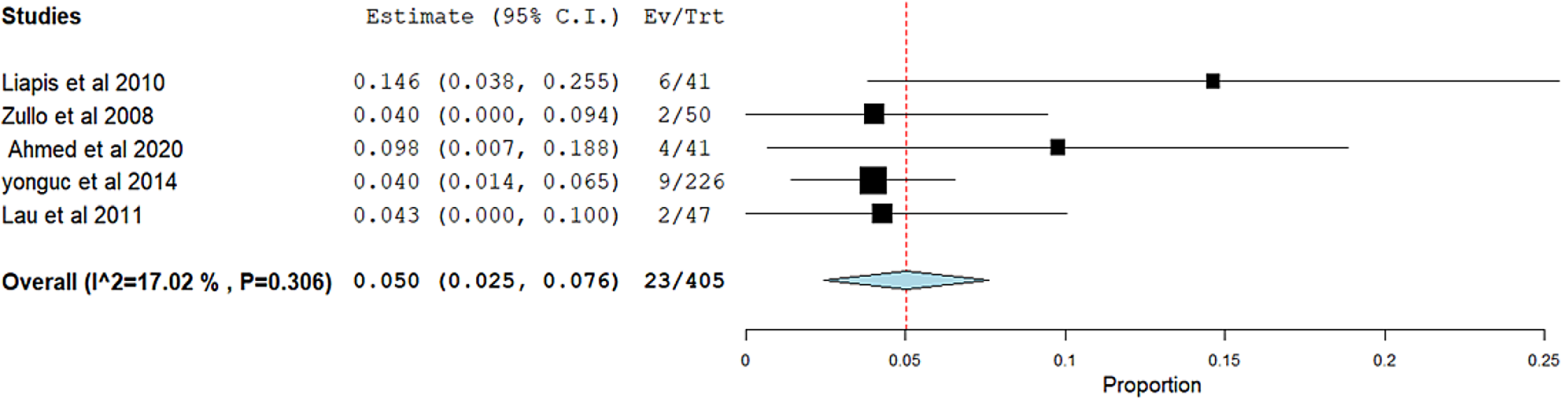
Meta-analysis forest plots for the incidence of Postoperative retention**:** Forest plot showing the pooled estimate (0.050; 95% CI 0.025 to 0.076) for Postoperative retention with low heterogeneity (I^**2**^ = 17.02%, p = 0.306). Squares represent individual studies; the diamond shows the overall effect size

**Postoperative retention:** The overall pooled effect estimate from five studies was 0.050 (95% CI 0.025 to 0.076). The studies demonstrated low heterogeneity, reflected by an I.^2^ of 17.02% and a corresponding p-value of 0.306. Figure 6

**Fig. 7:**
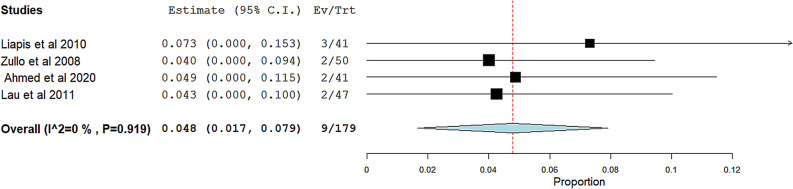
Meta-analysis forest plots for the incidence of Postoperative urinary tract infection**:** Forest plot showing the pooled estimate (0.048; 95% CI 0.017 to 0.079) for Postoperative retention with no heterogeneity (I^2^ = 0%, p = 0.919). Squares represent individual studies; the diamond shows the overall effect size

**Postoperative urinary tract infection**: The overall pooled effect estimate from four studies was 0.048 (95% CI 0.017 to 0.079). The analysis showed no heterogeneity, indicated by an I.^2^ of 0% and a corresponding p-value of 0.919. Figure 7

**Fig. 8:**
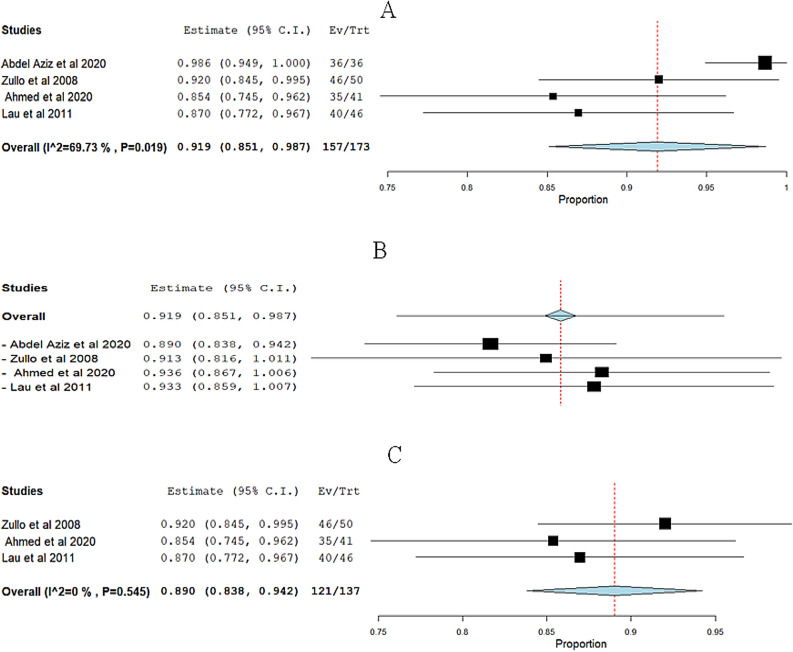
Meta-analysis forest plots for the Anatomical success of cystocele: **A** Pooled cure rate with heterogeneity (I^2^ = 69.73% %, p = 0.019). **B** Leave-one-out sensitivity analysis. **C** Pooled cure rate with heterogeneity (I^2^ = 0%, p = 0.545). Squares represent individual study estimates; diamonds show overall effect sizes

**Anatomical success of cystocele**: The overall pooled effect estimate from four studies was 0.919 (95% CI 0.851 to 0.987). The heterogeneity was with an I^2^ of 69.73% and a corresponding p-value of 0.019 Fig. 8A. In order to tackle the heterogeneity issue, we performed a leave-one-out sensitivity analysis Fig. 8B Excluding Abdel Aziz et al. 2020 [28] effectively solved the heterogeneity, resulting in the p-value for heterogeneity rising to 0.545 and reducing I^2^ to 0%. Figure 8C.

**Fig. 9:**
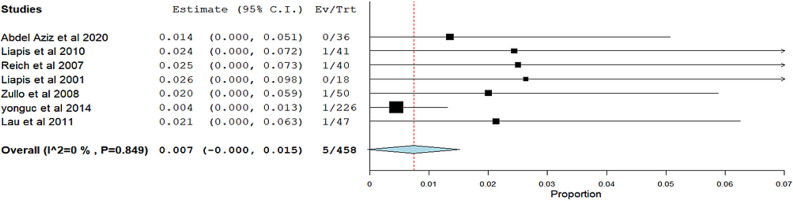
Meta-analysis forest plots for Tape Complications of mid-uretheral slings: Forest plot showing the pooled estimate (0.007; 95% CI 0.000–0.015) for tape complications with moderate heterogeneity (I^**2**^ = 0%, p = 0.849). Squares represent individual studies; the diamond shows the overall effect size

**Tape Complications**:The overall pooled effect estimate from seven studies was 0.007 (95% CI −0.000 to 0.015). The analysis showed no heterogeneity, indicated by an I.^2^ of 0% and a corresponding p-value of 0.849 Fig. 9

**De novo urge urinary incontinence**: Liapis et al. 2010 reported de novo urge urinary incontinence in 4 out of 41 patients [21]. Reich et al. 2007 found de novo urge urinary incontinence in 8 out of 40 participants [22]. Combining these, the overall percentage of de novo urge urinary incontinence across these studies is approximately 14.3% (12 out of 81 patients)[21, 22]. Additionally, Lau et al. 2011 observed de novo overactive bladder (OAB) symptoms in 4 out of 46 patients [30].

## Discussion

We analyzed data from the included studies, recording an objective cure rate for SUI of 90.7% (446/498) patients, an objective failure for SUI of 3.6% (10/186) patients, and a subjective cure rate for SUI of 82.5%)251/325) participants. We conducted a leave one out test by excluding Reich et al. as it has the shortest follow up period, un like the other included studies with a range of two to nine months. By leaving Reich et al., the objective cure rate for SUI became 89.5%, and the subjective cure rate became 77.6%. The anatomical success rate for cystocele was 91.9% (157/173) patients. After conducting the leave one out test and leaving Abdel Aziz et al., as the only study with a symptomatic grade 2 cystocele, the anatomical success rate for cystocele became 89%. Two of our included studies reported data at medium term follow-up periods, and log term follow up period (10, 13) years [21, 31–33].

At a long-term follow-up of 13 years an objective cure rate for SUI of 78% (32/41) was reported by Bakas et al. 2018 [32]. Montera et al. 2018. documented cumulative success rates for SUI of 92%, 84%, and 78% at 1, 5, and 10 years post-surgery, respectively [31].

Two hundreds and twenty nine patients in addition to 59 patients with initial synthetic MUS (SMUS) underwent a standard fascial pubovaginal sling (AF-PVS) surgery) in a prospective cohort study conducted by Parker et al. The primary AF-PVS group demonstrated an objective cure rate of 66.8% for SUI, with a subjective cure rate of 51.1% [34]. Additionally, 16.3% of patients in the primary AF-PVS group experienced de novo urgency [34]. The primary AF-PVS group exhibited an objective cure rate of 66.8% for SUI, with a subjective cure rate of 51.1% [29]. Additionally, 16.3% of patients in the AF-PVS group presented with de novo urgency. Within the scope of our analysis, de novo urge urinary incontinence was documented in 12 out of 81 patients, and de novo overactive bladder (OAB) symptoms were reported in 4 of 46 participants [21, 22, 30]. Postoperative retention was noted in 5% of 405 patients, compared to 3.1% in Parker et al.'s primary AF-PVS group [34].

According to Colombo et al., Burch colposuspension or anterior colporrhaphy alone have not efficaciously treated patients with SUI and clinically severe cystocele [35]. Paick et al. performed a logistic regression analysis and reported the factors influencing persistent SUI post-procedure. These factors included the following: severe cystocele grade, the type of procedure, comorbidities, and mixed urinary incontinence. They were considered high-risk factors. The authors stressed that, in order to enhance treatment outcomes, POP should be treated during SUI surgery. They also emphasized the need for careful evaluation of POP to ensure that the surgical approach is well-suited to the patient's condition [36].

Anterior colporrhaphy is intended to reinforce and firm the vaginal muscularis and adventitia layers that support the bladder, also referred to as the pubocervical, pubovesical, or endopelvic fascia [13]. In our review, the anatomical success rate of cystocele was 91.9% (95% CI 85.1% to 98.7%) during short- to medium-term follow-up. While Montera et al. 2018. reported cumulative cystocele success rates of 92%, 86%, and 82% at 1, 5, and 10 years, respectively, with 89% of patients achieving a cure for both SUI and cystocele [31]. Bakas et al. noted a success rate of 85% (35/41) at long-term follow-up of 13-years [32].

Different types of polypropylene mesh overlays were investigated for the treatment of anterior vaginal wall prolapse. The anatomical success rates for these overlays ranged from 75 to 100% [37]. A 2016 Cochrane review compared native tissue repairs with those augmented by biological grafts and mesh, finding that mesh repairs had lower rates of anatomical failure and prolapse perception at 1–3 years, but higher rates of reoperation and mesh-related complications. The authors concluded that while transvaginal mesh may be beneficial for patients at high risk of recurrence, its routine use in primary surgery is limited due to the associated morbidity, but there isn't any proof to support this up currently [38]. MUS with AC can be an effective option for treating of SUI accompanied by cystocele, demonstrating solid objective cure rates and good anatomical success for cystocele. Patients also report favorable subjective outcomes, with fewer postoperative complications.

This systematic review has several strengths. First, the paper follows the criteria that are currently in use for reporting meta-analyses and systematic reviews. We presented the most current and comprehensive summary of the available studies on interventions for female SUI associated with anterior vaginal wall prolapse grades 1–3, involving mid-urethral sling (MUS) with concomitant anterior colporrhaphy (AC). Most of the participants included in this systematic review had not undergone prior surgical intervention in the anterior compartment for prolapse or stress urinary incontinence. We included in the review two studies with long term follow up (ten and 13 years). We provided multiple outcomes. As well as we managed to solve the heterogeneity among included studies. Finally we hope to bridge a significant gap in the current literature.

However, we recognized many limitations. There is a lack of RCTs evaluating this approach so we managed to include only two RCTs one of them with some concerns [28]. As well as we had to include both prospective and retrospective cohorts that we could find, and one of them was with poor quality [29]. We also considered the possibility of conducting a sub-group analysis, but due to the small number of studies, it was not feasible. We had a limited sample size, in addition to the cure rate of SUI was not clearly separated into objective and subjective categories in a certain publication [28]. Another limitation of this study is that the GRADE ratings were lower due to indirectness and publication bias [26]. This was mainly due to the characteristics of the single-arm analysis. Finally, the inclusion of prospective cohorts, retrospective cohorts, and RCTs in the same analysis may introduce variability due to differing study designs. A sub-group analysis was not feasible due to the small number of studies.

Regarding research implications, there is a need for more rigorously designed prospective and randomized studies with larger sample sizes and extended follow-up periods to assess the benefits and drawbacks of mid-urethral sling (MUS) with concomitant anterior colporrhaphy (AC) in treating female stress urinary incontinence associated with grade 1–3 cystocele. In order to ensure the unbiased nature of the findings, research ought to have uniform, defined standards for concomitant continence and prolapse repair surgery.

## Conclusion

According to our review, MUS with AC is an efficient treatment option for treating SUI associated with grade 1–3 cystocele, showing substantial rates of both objective and subjective cure for SUI, as well as significant anatomical success in correcting cystocele. While we aimed to advance research on managing SUI with prolapse, additional rigorously conducted randomized studies with extended follow-up are needed to validate these findings and establish the optimal surgical approach.

## Data Availability

No datasets were generated or analysed during the current study.
